# Binding interaction of sodium benzoate food additive with bovine serum albumin: multi-spectroscopy and molecular docking studies

**DOI:** 10.1186/s13065-019-0615-6

**Published:** 2019-07-24

**Authors:** Jing Yu, Jian-Yi Liu, Wei-Ming Xiong, Xiao-Yue Zhang, Yue Zheng

**Affiliations:** 1 State Key Laboratory of Optoelectronic Materials and Technologies, Sun Yat-sen University, Guangzhou, 510275 China; 2 Micro&Nano Physics and Mechanics Research Laboratory, School of Physics, Sun Yat-sen University, Guangzhou, 510275 China; 3 Sino-French Institute of Nuclear Engineering and Technology, Sun Yat-sen University, Zhuhai, 519082 China

**Keywords:** Sodium benzoate, Bovine serum albumin, Multi-spectroscopy, Molecular docking modeling

## Abstract

Sodium benzoate (SB) is widely used as a preservative in food industry, and bovine serum albumin (BSA) is a major carrier protein similar to human serum albumin (HSA), the study of the binding between the two has great significance on human health. In this paper, we systematically investigated the binding of SB and BSA under the simulated physiological conditions combining with various common analytical methods, e.g., fluorescence, UV–vis absorption, synchronous fluorescence and circular dichroism (CD) spectra, as well as molecular docking method. The fluorescence quenching measurements were respectively carried out at 298 K, 303 K and 308 K using the Stern–Volmer method. The results reveal that ground state SB–BSA complex was formed within the binding constants from 2.02 × 10^4^ to 7.9 × 10^3^ M^−1^. Meanwhile, the negative values of Δ*H*^0^ (− 43.92 kJ mol^−1^) and Δ*S*^0^ (− 111.6 J mol^−1^ K^−1^) demonstrated that both the hydrogen binding interaction and van der Waals forces contributed to stabilizing the SB–BSA complex. The site marker competitive experiments show that the SB and BSA bound at site I. Furthermore, the experimental results of UV–vis absorption, synchronous fluorescence and CD spectra indicate that the binding of SB and BSA may change the conformation of BSA. In addition, the molecular docking experiment suggests that hydrogen bond was formed in the interaction between SB and BSA.

## Introduction

**Fig. 1:**
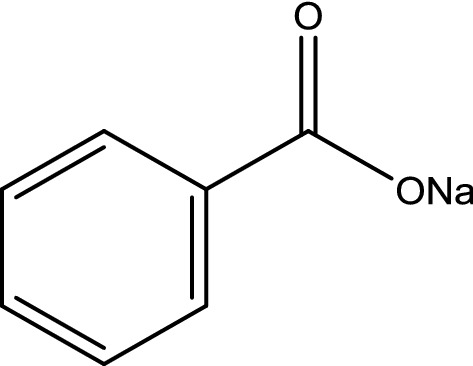
The chemical structure of sodium benzoate (SB)

As a recognized food-grade preservative, sodium benzoate (SB), whose structure is shown in Fig. 1, is widely used in the food, cosmetic, and pharmaceutical industries [1–3]. For example, in the food industry, SB is used in a variety of foods and beverages, such as salads, kimchi, carbonated beverages, jams, juices, and soy sauce, due to its effective inhibition of fungal and bacterial growth during storage [2, 4]. In addition, SB is also applicable in clinically practice and can to treat various diseases, such as urea cycle disorders, liver disease, multiple sclerosis, and early Alzheimer’s disease and Parkinson’s disease [5, 6]. Although SB as a preservative is generally recognized as safe (GRAS), its concentration is limited to 0.1% by the US Food and Drug Administration (FDA) [7]. In recent years, studies have shown that the organic form of SB is nontoxic, but its synthetic form is toxic to organisms at chronic doses [8]. Furthermore, it has been reported that SB may damage mitochondrial DNA [9]. Nevertheless, the results of these investigations remain controversial. The interaction between SB and biomacromolecules requires a more in-depth research.

The effect of SB on health can be explored by studying the interaction between SB and Serum albumin (SA). SA, the most abundant translocator protein in blood circulation [10, 11], has several critical physiological functions, for example, the maintenance of the colloidal osmotic blood pressure, and the transportation of various endogenous and exogenous compounds [12, 13]. Bovine serum albumin (BSA) is not only widely used in biomedical and pharmaceutical applications, but also widely utilized as a ligand-biological model to study the interactions between small molecules and globular proteins, due to its high stability, low-cost, versatile ligand-binding properties, medical importance and high structural homology with human serum albumin (HSA) (approximately 76%) [14, 15]. BSA is a single-chain globular protein consisting of 583 amino acid residues, forming 17 disulfide bonds [16]. In addition, BSA contains two major specific ligand-binding sites located in the hydrophobic cavities in sub-domains II-A and III-A, which are also known as Sudlow’s site I and Sudlow’s site II, respectively [17]. Meanwhile, previous works have demonstrated that the durability and toxicity of chemicals have huge influence on the structure of BSA because of interaction effect [18]. Moreover, several studies have shown that secondary structure of BSA changes upon binding with small molecules [19–21]. Therefore, investigating the interaction between BSA and chemicals, especially small molecules is of great significance.

In this work, the interaction between SB and BSA was studied by employing multi-spectroscopic methods and molecular docking (MD) modeling. Fluorescence spectroscopy was first used for understand the binding mechanism, and quenching mechanism, binding constant, mode and site were analyzed. Then, UV–vis absorption, synchronous fluorescence and CD spectra were employed to determine the conformation and structure of BSA when SB was binding. Furthermore, to further interpret the experimental findings, molecular docking modeling was used to explore the molecular graph of the binding interaction.

## Experimental

### Chemicals and reagents

All chemicals used in this experiment, including BSA (lyophilized powder, Mw ≈ 66.2 kDa, Sigma) and sodium benzoate (Sinopharm), are of high purity. Thus, no further purification is required after purchase. PBS buffer solution (20 mM, pH = 7.40) was prepared with the ultrapure water (*ρ *= 18.2 MΩ cm). Then, BSA solution was prepared using PBS as a solvent for the spectral experiments. Additionally, all solutions were stored in the dark environment with a low temperature of 4 °C before using.

### Measurements of the fluorescence spectra

Fluorescence experiments were taken out with the RF-5301 (Shimadzu, Japan) fluorescence spectrophotometer. Firstly, BSA solution (1 × 10^−6^ M) was added to a quartz cell with size of 1.0 cm. An equal amount of BSA was added to the reference solution to eliminate the absorbance of BSA itself, and the absorbance of PBS buffer was subtracted through base line correction. Then, SB of different concentrations from 0 to 8 × 10^−6^ M was gradually dropped into the BSA using a microsyringe. The transportation of various molecules and materials in the blood circulatory system are regulated by albumin. Some of the small molecules entered into the blood are reversibly bound to plasma proteins, forming binding molecules. While those do not bind are free molecules. When the concentration of free molecules decreases, some of the binding molecules dissociate into free molecules, thus, they are always in dynamic equilibrium [22]. In our experiment, SB and BSA were let stand for 5 min to reach dynamic equilibrium [23]. During the fluorescence measurement, the slit width was set at 10 nm/10 nm, the excitation wavelength was 285 nm, and the scanning range of the fluorescence emission spectrum was 300–450 nm.

### Measurements of the synchronous fluorescence

The synchronous fluorescence of SB–BSA was measured using the same concentrations of the mixture solutions as in the fluorescence quenching measurements, but at room temperature. Spectra were recorded at Δλ = 15 nm and 60 nm, which showed the tyrosine residue and tryptophan residue characteristics of the BSA.

### Measurements of UV–vis spectra

The UV–vis absorption spectra were obtained by a UV-3600 (Shimadzu, Japan) spectrophotometer. The concentration of BSA was kept at 1 × 10^−6^ M, while the concentration of the added SB ranged from 0 to 8 × 10^−6^ M, each time with an increase of 2 × 10^−6^ M. The absorption spectra of the BSA between 230 and 330 nm were recorded in a 1 cm quartz absorption cell.

### Measurements of CD spectra

CD spectra were recorded using the spectropolarimeter of JASCO J-810 and a 1.0 cm quartz absorption cell. Note that the spectrum of a cell only with PBS buffer solution was firstly measured as background signal to remove the influence of PBS buffer solution.

### Molecular docking

Molecular docking simulation was employed to study the molecular interaction between BSA and SB using Auto Dock Vina, an open-source software with significantly fast dock running speed and high molecular docking accuracy [24]. To prepare the protein and ligand molecules for docking study, BSA crystal structure was first retrieved from the Protein Data Band (PDB ID: 3V03) (http://www.rcsb.org/structure/3V03), and then loaded on AutoDock Tools to remove additional molecules, e.g., all the water molecules. Next, Polar hydrogens and Gasteiger charges were added, respectively. The structure of SB was prepared by drawing the 2D chemical structure using ChemOffice and further optimized based on MM2 force field implemented using Chem3D. After that, the structurally optimized BSA and SB were employed to conduct molecular docking simulation. During the simulation, the size of the grid box along x-, y- and z-directions were all set at 18 Å and the grid spacing 1 Å. The grid box center was set at (88.537, 24.797, 13.111). The energy range and values were set at 4 kcal/mol and 100, respectively.

## Results and discussion

### Fluorescence spectral analysis of the interactions involving SB with BSA

#### Fluorescence quenching of BSA

**Fig. 2:**
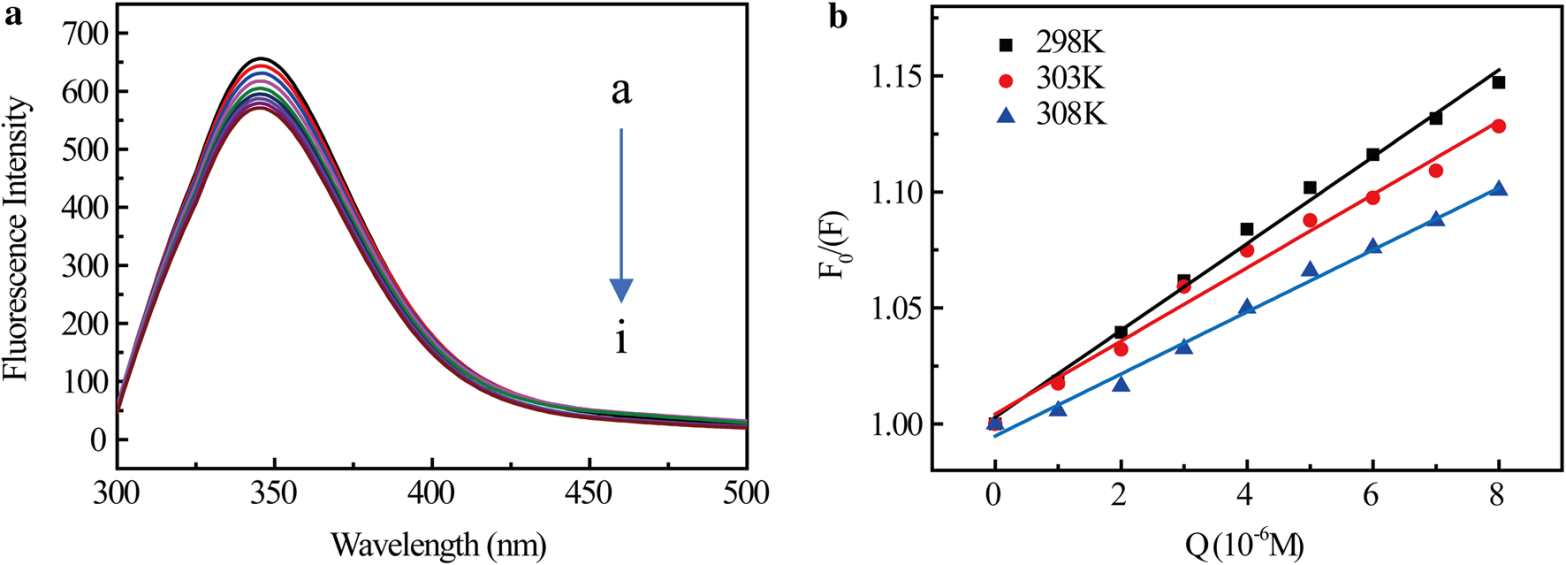
Fluorescence spectra of BSA solution (1 × 10^−6^ M) in the absence and presence of different concentrations of SB (T = 298 K, pH = 7.40, λ ex = 285 nm). **a** From a to i, the SB concentration increased from 0 to 8.0 × 10^−6^ M, with a step size of 1.0 × 10^−6^ M. **b** Quenching effect of Stern–Volmer on BSA induced by SB

Tryptophan (Trp), tyrosine (Tyr) and phenylalanine (Phe) residues are three main amino acids that can make protein generate endogenous fluorescence [25]. The major contribution to the changed fluorescence of BSA is from the environmentally-sensitive tryptophan (Trp) moiety [26]. The fluorescence emission spectra of BSA with various concentration of SB are shown in Fig. 2a. When excited at 285 nm, BSA had a characteristic band at around 344 nm. Furthermore, when the concentration of the added SB increased from 0 to 8 × 10^−6^ M, the fluorescence intensity of BSA decreased significantly, indicating that the environment around the Trp residues of BSA varied with the addition of SB. Therefore, it can be inferred that there is a binding interaction between SB and BSA, and the binding site is located near the Trp residue [27].

The reaction temperatures for SB–BSA system were maintained at 298 K, 303 K, and 308 K, respectively. The fluorescence quenching data are analyzed by the Stern–Volmer equation [28]:

1 $$\frac{{F_{0} }}{F} = 1 + K_{\text{SV}} \left[ {\text{Q}} \right]{ = }1{ + }k_{\text{q}} \tau_{0} \left[ {\text{Q}} \right]$$

where *F* and *F*_0_ are the fluorescence intensity of BSA with and without the quencher, i.e., SB, respectively. *K*_SV_ is the Stern–Volmer quenching constant with the unit being M^−1^, and [Q] is the concentration of the quencher. *k*_q_ is the quenching rate constant of BSA. *τ*_0_ is the average fluorescence lifetime of BSA in the excited state without the quencher (the order of magnitude is 10^−8^) [28]. *K*_SV_ and *K*_q_ value of BSA triggered by SB at different temperatures can be determined by calculating the slope of the curve, as shown in Fig. 2b. The values of the parameters *K*_SV_, *k*_q_ and R at different temperatures are listed in Table 1. It can be seen from the results that *K*_SV_ decreases from 1.87 × 10^4^ to 1.34 × 10^4^ M^−1^ as the temperature increases from 298 to 308 K. Moreover, the values of *k*_q_ at various temperatures are all in the order of 10^12^ M^−1^ s^−1^, which are much larger than the maximum diffusion collision quenching rate constant (2.0 × 10^10^ M^−1^ s^−1^), indicating that the SB-trigger BSA quenching process is static rather than dynamic.

**Table 1:** Stern–Volmer Constants of Stern-Volmer quenching (K_SV_) and bimolecular quenching rate (K_q_) at tested temperatures

*T* (K)	*K*_SV_ (10^4^ M^−1^)	*K*_q_ (10^12^ M^−1^ S^−1^)	R
298	1.87	1.87	0.99327
303	1.58	1.58	0.99302
308	1.34	1.34	0.99579

R represents correlation coefficient

#### Interaction parameters and binding model for SB–BSA complex

The binding constants (*K*_b_) and binding site (*n*) of SB–BSA complex is calculated using the following formula [29]:

2 $$\lg \frac{{(F_{0} - F)}}{F} = \lg K_{\text{b}} + n\lg \left[ {\text{Q}} \right]$$

**Fig. 3:**
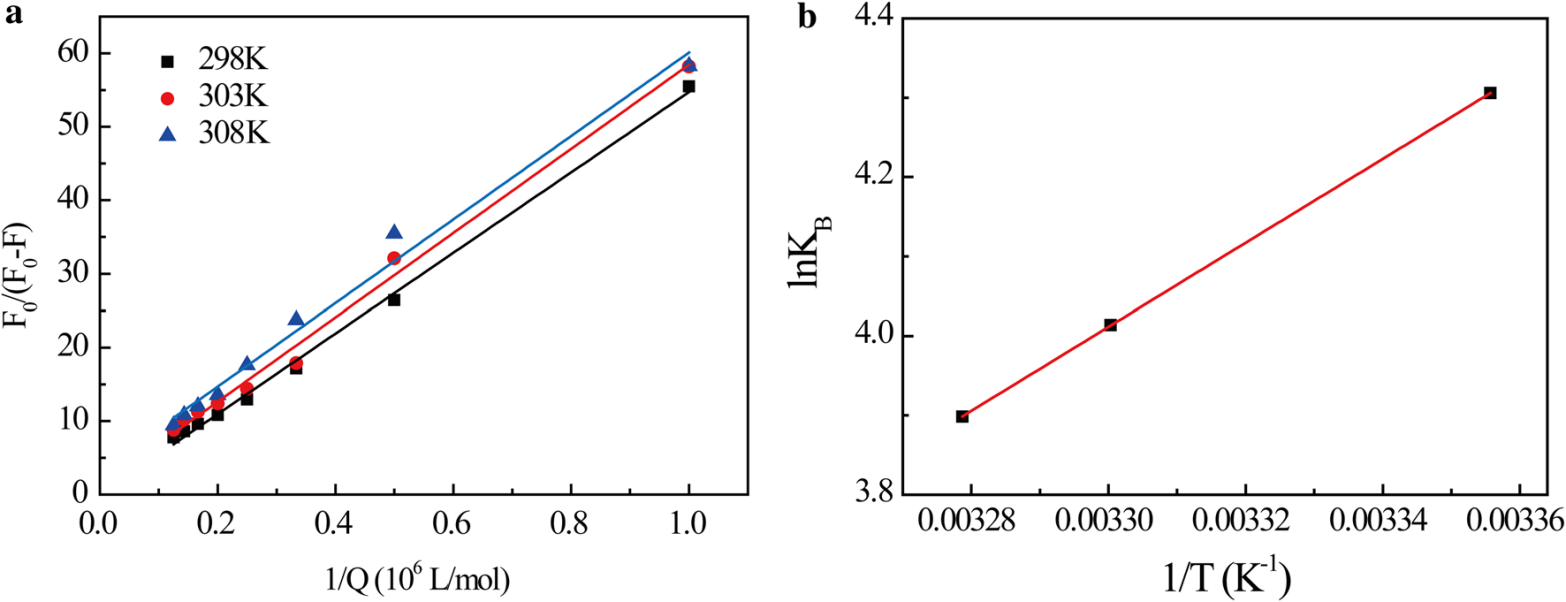
**a** Lineweaver–Burk plot of SB–BSA at different temperatures. **b** van’t Hoff plot of SB–BSA binding

According to the Eq. (2), *K*_b_ and n can be calculated from the curve of log[(F_0_−F)/F] versus log[Q], as shown in Fig. 3a. The calculated results are summarized in Table 2. These results show that within the temperature range studied, the value n of SB–BSA complex is close to 1, indicating that BSA has a single high affinity binding site for SB. *K*_b_ is calculated to be approximately 10^4^, indicating strong binding interactions between SB and BSA. It is also found that as temperature increases, *K*_b_ value decreases, suggesting that the stability of SB–BSA complex decreases with the increasing of temperature.

**Table 2:** Calculated parameters of SB–BSA complex at different testing temperatures at pH 7.4

*T*(K)	*K*_b_ (10^4^ M^−1^)	*n*	R	Δ*G*^0^ (KJ/mol)	Δ*S*^0^ (J mol^−1^ K^−1^)	Δ*Η*^0^ (KJ/mol)
298	2.02	1.00	0.9973	− 9.35	− 111.6	− 43.92
303	1.03	0.96	0.9933	− 8.78	− 111.6	− 43.92
308	0.79	0.95	0.9971	− 8.12	− 111.6	− 43.92

*K*_b_, *n*, *G*^0^, *S*^0^ and *Η*^0^ are binding constant, binding site, Gibbs free energy, thermodynamic enthalpy and entropy, respectively. R represents correlation coefficient

To better understand the binding between BSA and SB, the van’t Hoff Eq. (3) was used to calculate the thermodynamic enthalpy (H_0_) and entropy (S_0_) of BSA and SB complexation.

3 $$\ln K_{\text{b}} = - \frac{{\Delta H^{0} }}{RT} + \frac{{\Delta S^{0} }}{R}$$

As shown in Fig. 3b, the curves of ln*K*_b_ and 1/T were used to determine the thermodynamic parameters of SB–BSA complex at three different temperatures, i.e. 298 K, 303 K, and 308 K. Once the H_0_ and S_0_ values are determined, the variation in Gibbs free energy (G_0_) can be calculated by the following standard Eq. (4).

4 $$\Delta G^{0} = \Delta H^{0} - T\Delta S^{0}$$

Here, the binding constant values at three different temperatures, i.e. ΔG_0_, ΔH_0_ and ΔS_0_, are listed in Table 2. The negative value of ΔG_0_ indicates that the interaction process between SB and BSA is spontaneous. And the positive H_0_ and S_0_ values indicate that hydrogen bonding and van der Waals interactions play a major role in the binding of the chemical to the protein [30, 31].

#### Combination of fluorescent probes

**Fig. 4:**
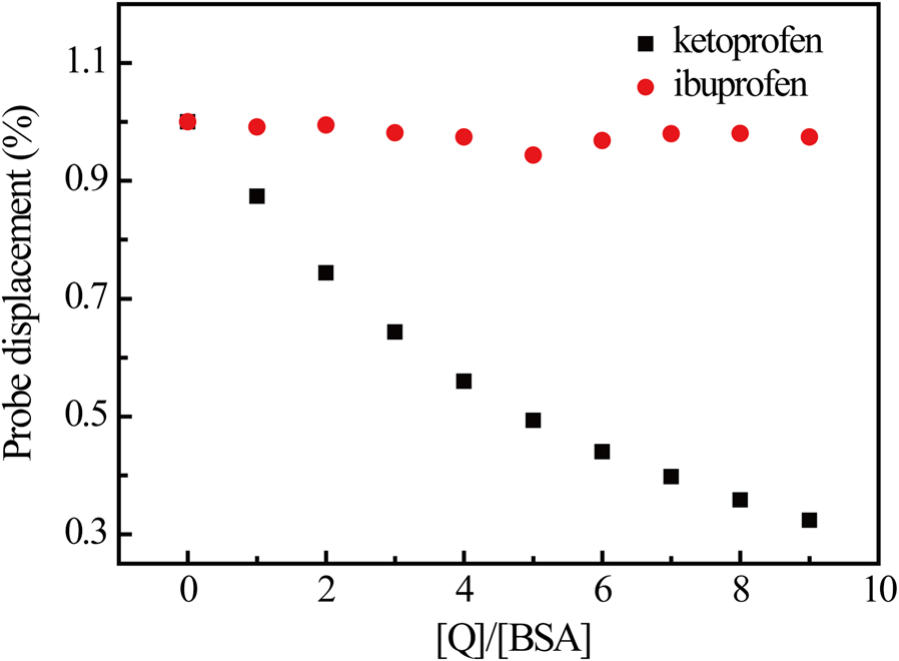
The effect of site probe on SB–BSA fluorescence. [SB] = 5.0 × 10^−5^ M, [BSA] = 1.0 × 10^−5^ M. [Q]: filled red circle, ibuprofen; filled black square, ketoprofen

To determine the displacement percentage of the fluorescent combination probe, according to the method introduced by Sudlow et al., the fluorescence markers for distinct binding sites were chosen: ketoprofen for site I and ibuprofen for site II [32]. And the following equation is also adopted.

5 $${\text{Probe displacement }}\left( \% \right) \, = {\text{ F}}_{ 2} /{\text{F}}_{ 1} \times { 1}00$$

where F_1_ and F_2_ represent the fluorescence intensity of the SB–BSA system in the absence and presence of the probe, respectively. As shown in Fig. 4. The increase in ketoprofen concentration results in significant decrease in the fluorescence intensity of the SB–BSA system. However, the increase in ibuprofen concentration has little impact on the fluorescence intensity of BSA. Therefore, SB and BSA are supposed to bind at site I [33].

### Conformational change of BSA

#### Analysis of the UV–visible absorption spectra

**Fig. 5:**
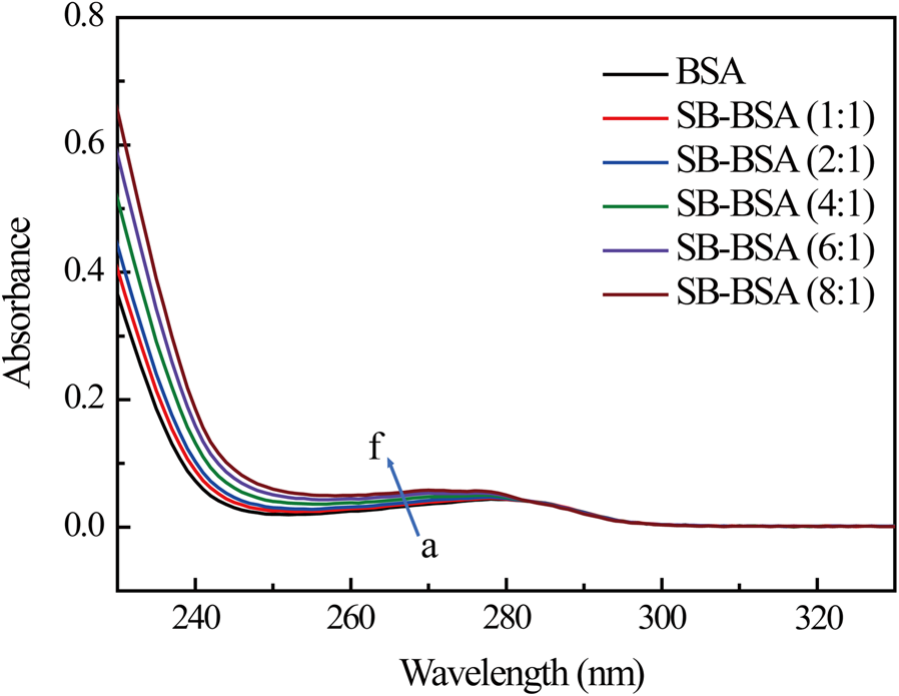
The UV–Vis absorption spectra of BSA in the absence and presence of different concentrations of SB. The BSA concentration was 1.0 × 10^−6^ M; while the SB concentration increased from 0 to 8.0 × 10^−6^ M, with a step size of 2.0 × 10^−6^ M from a to f

UV absorption measurement, a very simple and effective method, is often used to observe the formation process and the change in the conformation of protein during the its binding interaction between with small molecule. The UV spectra of SB–BSA complex are shown in Fig. 5. All SB–BSA complex have absorption bands. The absorption band around 280 nm is the result of the π–π* transition of aromatic amino acids (Trp, Tyr, and Phe) [34]. Adding SB (0–8.0 × 10^−6^ M) to the BSA solution enhances the absorption intensity (0.044–0.0556), and the maximum wavelength shows a slight blue shift at around 280 nm. The UV absorption intensity increases with the increase of SB concentration, indicating that complex is formed by SB and the amino acid residues of BSA.

#### Synchronous fluorescence spectroscopy

**Fig. 6:**
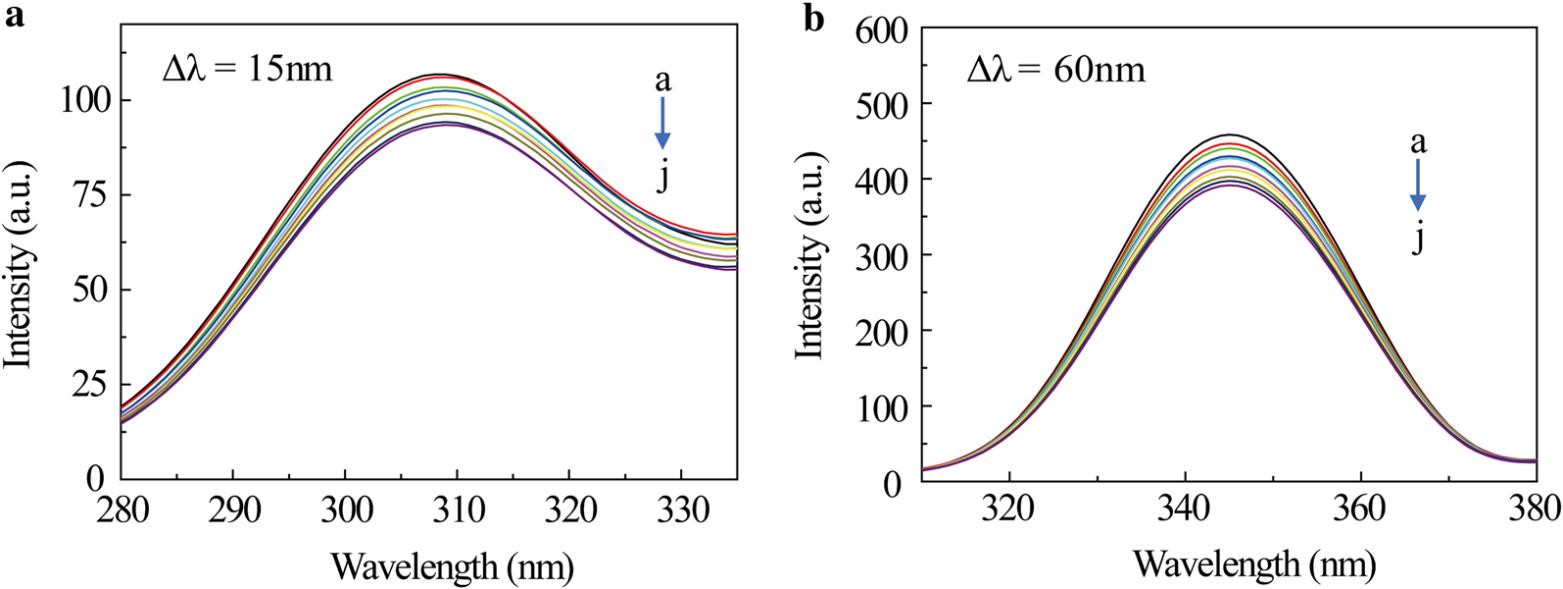
The synchronous fluorescence spectra of SB–BSA. **a** Δλ = 15 nm; **b** Δλ = 60 nm. [BSA] = 1.0 × 10^−6^ M. From a to j, the concentration of SB was varied from 0 to 9.0 × 10^−6^ M with a step of 1.0 × 10^−6^ M

The synchronous fluorescence spectroscopy is a useful tool for obtaining information around chromophore microenvironment. Usually, the shift of the λem represents the alteration of the polarity of the amino acid environment (particularly Tyr or Trp residues) [35]. The synchronous fluorescence spectra were carried out for investigating structural change in BSA with SB addition (Fig. 6). The fluorescence spectra characteristic of tyrosine and tryptophan residues are shown in Fig. 6a (Δλ = 15 nm) and Fig. 6b (Δλ = 60 nm), respectively [36]. It is obvious that the fluorescence intensity of the tryptophan residues are much stronger than that of tyrosine residues in Fig. 6. And no significant change is found in the fluorescent emission peak position of both tyrosine and tryptophan residues. Thus, the addition of SB did not rearrange the microenvironment of the tyrosine and tryptophan residues in BSA.

#### Analysis of CD spectra

**Fig. 7:**
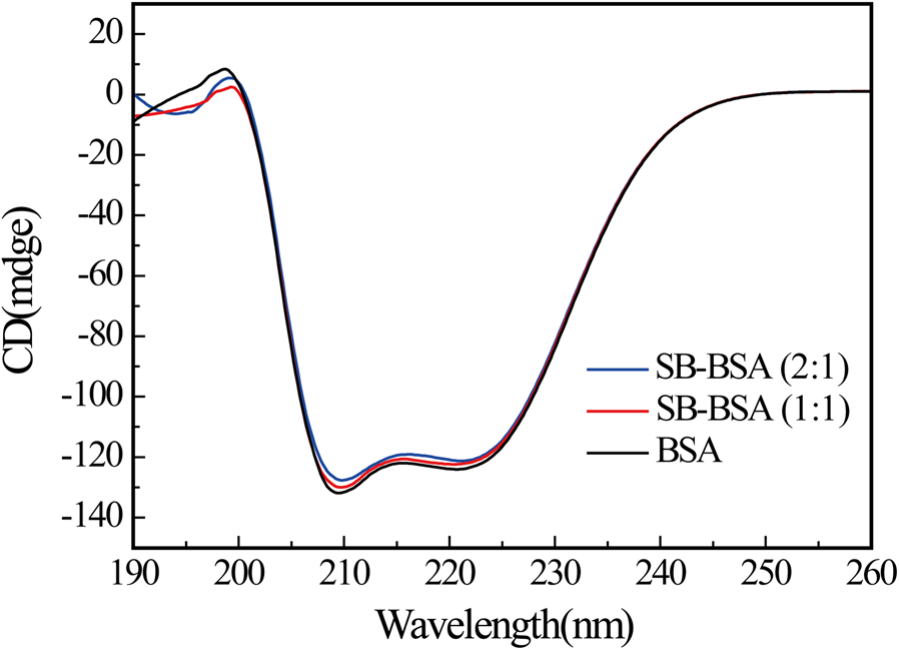
CD spectra of the SB–BSA system at room temperature. The concentration of BSA was 1.0 × 10^−6^M, whereas the concentration of SB were 1.0 × 10^−6^M (red line) and 2.0 × 10^−6^M (blue line), respectively

To further explore whether SB can induce conformational change in BSA, CD spectroscopy experiments were performed. The two negative bands at 208 nm (π–π* transition) and 222 nm (n-π* transition) in CD spectrum of free BSA are characteristic of the protein α-helical structure [37], whose content can be estimated by [38]:

6 $${\text{a-}}{\text{helix }}\left( \% \right) \, = \, \left[ {\left( {{-}{\text{ MRE}}_{ 20 8} {-}{ 4}000} \right) \, / \, \left( { 3 3000 \, {-}{ 4000}} \right)} \right] \, \times { 100}$$

where MRE_208_ is the MRE value observed at 208 nm, 4000 is the MRE value of the β shape and random coil conformation at 208 nm, and 33000 is the MRE value of the pure α-helix at 208 nm. The MRE_208_ value used to indicate the change in secondary structure of BSA determined by [38]:

7 $${\text{MRE}}_{ 20 8} = CD\left( {\text{m deg}} \right)_{ 20 8} /\left( { 10 \, \times n \times l \times C_{\text{p}} } \right)$$

where *n* is the number of amino acid residues (583 for BSA). *l* is the cell path length, and *C*_p_ is the molar concentration of BSA. When SB:BSA ratio increases from 0 to 2:1, the band intensities of the CD spectra have a slight decrease, whereas the peaks positions remain unchanged, as shown in Fig. 7. The results demonstrate that the secondary structure of BSA has a partial change from α-helical content. The α-helical content decreased from 62.7% of free BSA to 61.88% (SB:BSA = 1:1) and 60.24% (SB:BSA = 2:1). Based on these analyses, we conclude that the addition of SB alters the secondary structure of BSA, resulting in a decrease in α-helical content, which is nonetheless still dominant in the secondary structure.

#### Molecular docking

**Fig. 8:**
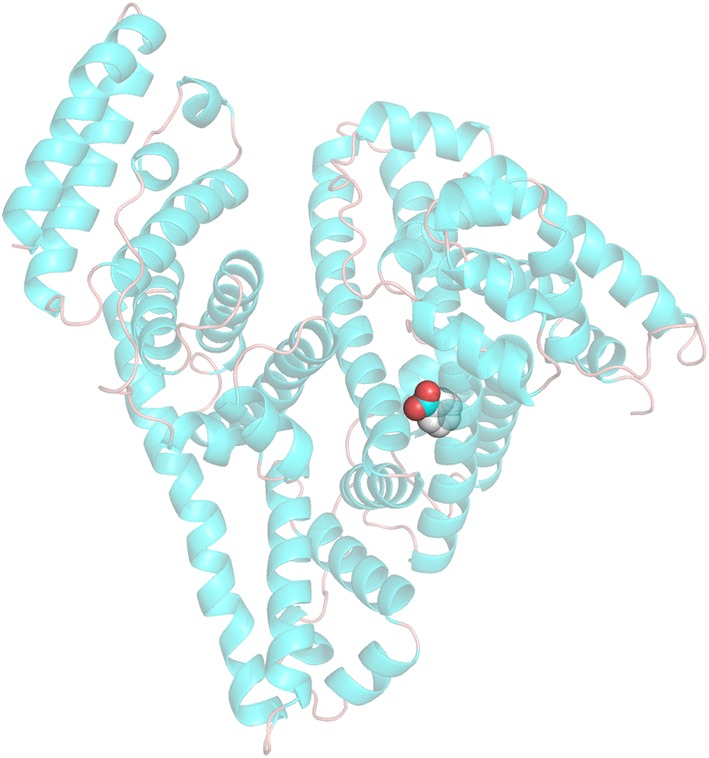
The docking conformation of SB–BSA complex and the lowest binding free energy

**Fig. 9:**
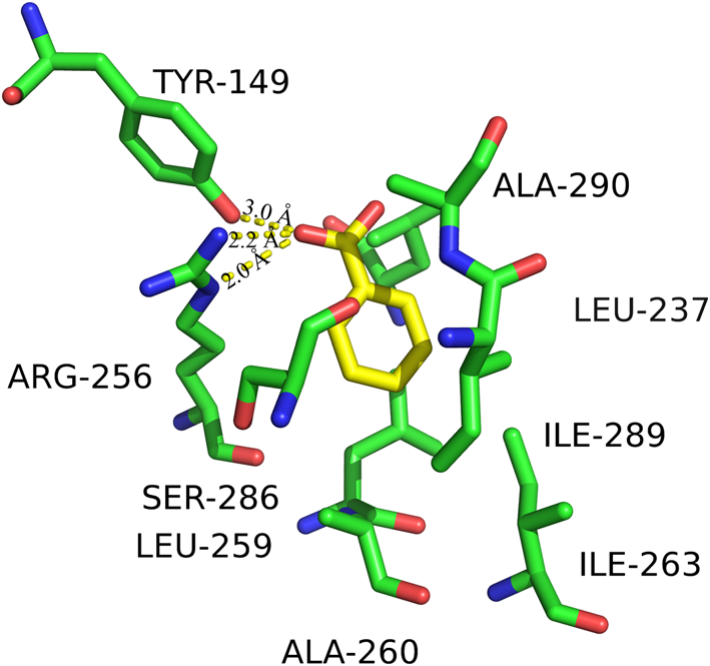
The interaction between SB and adjacent residues of BSA. The hydrogen bonds formed in the SB–BSA system are marked as yellow dotted lines

To further elucidate SB–BSA binding interaction, molecular docking was used to simulate the molecular interaction between BSA and SB. The simulated result of the predominate configuration of SB–BSA complex is plotted in Fig. 8, where the binding energy is the lowest. Moreover, as shown in Fig. 9, the binding results indicate that SB is very close to the amino acid residues Tyr149, Leu237, Arg256, Leu259, Ala260, Ile263, Ser286, Ile289, and Ala290 at site I in subdomain IIA. The hydrogen bonding between SB and BSA (Tyr149, ARG256 with a bond length of 3.0 Å, 2.2 Å and 2.0 Å) is also responsible for maintaining the stability of the complex. Here, note that the calculated combined Gibbs free energy is − 5.7 kcal/mol (− 23.8 kJ/mol) and binding constant (K_b_) is about 1.45 × 10^4^ mol/L, which is quite different with our experimental result (ΔG = − 935 kJ/mol and K_b_ = 2.2 × 10^4^ mol/L). This may be caused by the difference between X-ray crystal structure of BSA and its solution state in the aqueous system.

## Conclusion

In this study, we combined the multi-spectroscopic methods and molecular docking modeling to systematically investigate the interaction between SB and BSA. The multi-spectroscopic data show that the quenching of BSA binding with SB was caused by the formation of SB–BSA complex. The negative values of Δ*H*^0^ and Δ*S*^0^ demonstrated that hydrogen bonding and van der Waals forces contribute to make the SB–BSA complex stable. The negative values of Δ*G* indicate that the interaction process was spontaneous. Site marker competitive experiments show that SB and BSA bound at site I. In addition, the amino acid microenvironments and the secondary structure of BSA were altered by the addition of SB, as shown in UV–vis absorption, synchronous fluorescence spectroscopy and CD spectra data. Furthermore, molecular docking studies provided some valuable information on the interaction between SB and BSA and structural stability of their complex.

## Data Availability

All data and material analyzed or generated during this investigation are included in this published article. The raw data can be requested from email of JY: 95634941@qq.com.

## Abbreviations

SB: sodium benzoate
BSA: bovine serum albumin
HSA: human serum albumin
CD: circular dichroism
GRAS: generally recognized as safe
FDA: Food and Drug Administration
SA: serum albumin
MD: molecular docking
